# Design of a phase III multicenter trial to evaluate the efficacy of the RTS,S/AS01 malaria vaccine in children across diverse transmission settings in Africa

**DOI:** 10.1186/1475-2875-10-224

**Published:** 2011-08-04

**Authors:** Amanda Leach, Johan Vekemans, Marc Lievens, Opokua Ofori-Anyinam, Conor Cahill, Seth Owusu-Agyei, Salim Abdulla, Eusebio Macete, Patricia Njuguna, Barbara Savarese, Christian Loucq, W Ripley Ballou

**Affiliations:** 1 GlaxoSmithKline Biologicals, Wavre, Belgium; 2 Kintampo Health Research Centre, Ghana Health Service, Kintampo, Ghana; 3 Ifakara Health Institute, Ifakara, Tanzania; 4 Centro de Investigação em Saude de Manhiça, Manhiça, Mozambique; 5 KEMRI-Wellcome Trust Research Programme, Kisumu, Kenya; 6 PATH Malaria Vaccine Initiative, Washington, USA

## Abstract

**Background:**

GlaxoSmithKline Biologicals and the PATH Malaria Vaccine Initiative are working in partnership to develop a malaria vaccine to protect infants and children living in malaria endemic regions of sub-Saharan Africa, which can be delivered through the Expanded Programme on Immunization. The RTS,S/AS candidate vaccine has been evaluated in multiple phase I/II studies and shown to have a favourable safety profile and to be well-tolerated in both adults and children. This paper details the design of the phase III multicentre efficacy trial of the RTS,S/AS01 malaria vaccine candidate, which is pivotal for licensure and policy decision-making.

**Methods:**

The phase III trial is a randomized, controlled, multicentre, participant- and observer-blind study on-going in 11 centres associated with different malaria transmission settings in seven countries in sub-Saharan Africa. A minimum of 6,000 children in each of two age categories (6-12 weeks, 5-17 months) have been enrolled. Children were randomized 1:1:1 to one of three study groups: (1) primary vaccination with RTS,S/AS01 and booster dose of RTS,S/AS01; (2) primary vaccination with RTS,S/AS01 and a control vaccine at time of booster; (3) primary vaccination with control vaccine and a control vaccine at time of booster. Primary vaccination comprises three doses at monthly intervals; the booster dose is administered at 18 months post-primary course. Subjects will be followed to study month 32. The co-primary objectives are the evaluation of efficacy over one year post-dose 3 against clinical malaria when primary immunization is delivered at: (1) 6-12 weeks of age, with co-administration of DTPwHepB/Hib antigens and OPV; (2) 5-17 months of age. Secondary objectives include evaluation of vaccine efficacy against severe malaria, anaemia, malaria hospitalization, fatal malaria, all-cause mortality and other serious illnesses including sepsis and pneumonia. Efficacy of the vaccine against clinical malaria under different transmission settings, the evolution of efficacy over time and the potential benefit of a booster will be evaluated. In addition, the effect of RTS,S/AS01 vaccination on growth, and the safety and immunogenicity in HIV-infected and malnourished children will be assessed. Safety of the primary course of immunization and the booster dose will be documented in both age categories.

**Conclusions:**

This pivotal phase III study of the RTS,S/AS01 candidate malaria vaccine in African children was designed and implemented by the Clinical Trials Partnership Committee. The study will provide efficacy and safety data to fulfil regulatory requirements, together with data on a broad range of endpoints that will facilitate the evaluation of the public health impact of the vaccine and will aid policy and implementation decisions.

**Trial registration:**

Clinicaltrials.gov NCT00866619

## Background

The past decade has seen unparalleled advances in the fight against malaria, and numerous public and private organizations are contributing hundreds of millions of dollars to malaria infection and disease research [1,2]. Malaria control interventions, including the use of long-lasting insecticide-treated nets and artemisinin-based combination treatment, have been broadly implemented [1], with some countries recently reporting an associated fall in malaria incidence [3]. Nevertheless, malaria continues to impose a considerable burden of morbidity and mortality, most significantly in young children, and reducing this burden in this population is, therefore, a public health priority in sub-Saharan Africa [4,5].

A safe and affordable vaccine would be a valuable addition to existing control measures. GlaxoSmithKline (GSK) Biologicals has been working towards the development of a safe and effective malaria vaccine for more than 20 years and has developed a candidate *Plasmodium falciparum *malaria vaccine, RTS,S/AS01, which is currently in phase III clinical trials in infants and children living in malaria-endemic regions of sub-Saharan Africa [6]. It is intended that the vaccine will be delivered through the Expanded Programme on Immunization (EPI) to leverage the vaccine delivery systems used to routinely administer immunizations to young children.

The candidate malaria vaccine targets the pre-erythrocytic stage of the *P. falciparum *parasite. It contains the RTS,S antigen and is formulated with a novel proprietary Adjuvant System (AS). Clinical trials of the vaccine formulated with closely related Adjuvant Systems - AS01 or AS02 - have been conducted, and the RTS,S/AS01 formulation has been selected for phase III development based on comparative clinical studies [7,8]. AS01 is composed of liposomes and the immunomodulatory molecules, 3-*O*-desacyl-4'-monophosphoryl lipid A (MPL) and QS21 [9].

A series of phase II clinical trials have been conducted to determine the safety, immunogenicity and efficacy of the RTS,S/AS vaccine in the target population of children at high risk of the disease. A proof-of-concept study in children aged 1-4 years in Mozambique showed that the RTS,S/AS02 vaccine was well-tolerated, with a vaccine efficacy of 35% against clinical malaria and 49% against severe malaria over 18 months [10,11]. Subsequent studies in infants have shown that the vaccine is well-tolerated and immunogenic in infants from 6 weeks of age, and can be successfully integrated into the EPI schedule [12,13]. Phase II studies have estimated the efficacy of the RTS,S/AS01 vaccine against clinical malaria to be 53% over eight months in 5-17 month old children and 59% over 17 months in 6-12 week old infants [14,15]. At the end of phase II, a pooled analysis of all paediatric safety data was conducted to support the progression of the RTS,S/AS candidate vaccine into large scale phase III clinical testing in Africa. Analysis of the extensive safety database of RTS,S/AS confirmed the favourable safety profile of the vaccine in children and infants living in malaria endemic regions in sub-Saharan Africa [Vekemans, Guerra, Lievens, Benns, Lapierre, Leach, Verstraeten: Pooled safety analysis of paediatric phase II RTS,S/AS malaria candidate vaccine trials, submitted].

The licensure claim of vaccine efficacy will be based principally on a large phase III clinical trial. This paper describes the overall design of the phase III multicentre efficacy study of the candidate RTS,S/AS01 malaria vaccine. The aims of this study are two-fold. Firstly, it will provide pivotal efficacy and safety data to support regulatory approval of the vaccine by the European Medicines Agency (EMA) and African national regulatory authorities and to facilitate pre-qualification by the World Health Organization (WHO). Secondly, it includes a broad range of endpoints that will allow assessment of the full public health impact of the vaccine. This information will be required to support a recommendation by the WHO and implementation decisions by local policy makers, and is key to ensure uptake of the vaccine following licensure [16]. Measures of vaccine efficacy on various disease endpoints in the phase III study may be utilized in an assessment of health economics. Additional data to support the full health economic evaluation are being collected in ancillary studies, such as assessment of quality of life, subject preferences, the measurement of resource utilization and direct and indirect costs, details of which will be described in separate publications.

The population of the phase III trial mirrors as closely as possible the population of children who usually attend EPI visits. Low-birth-weight infants, malnourished children, and HIV-infected children were eligible. For safety reasons, those that were critically sick were excluded: in particular any child who required hospital admission or had an advanced stage of HIV disease (WHO classification grade 3 or 4). A dedicated phase III study will assess safety and immunogenicity in children exposed to HIV (NCT01148459). Two further studies will evaluate the safety and immunogenicity of RTS,S/AS01: the first in co-administration with rotavirus and *Streptococcus pneumoniae *vaccines, which are expected to become part of the EPI program in the near future, and the second with three lots of RTS,S/AS01 vaccine in order to demonstrate lot-to-lot consistency.

Clinical development of the RTS,S/AS01 vaccine is undertaken in a public private partnership between GlaxoSmithKline and the PATH Malaria Vaccine Initiative (MVI), which receives funding from the Bill and Melinda Gates Foundation. The trial is also supported by the Malaria Clinical Trials Alliance (MCTA), an African-led organization that aims to build capacity and share best practice for the conduct of clinical trials. This multi-centre efficacy trial was designed by the Clinical Trials Partnership Committee (CTPC), which has membership representing each of the academic institutions participating in trial conduct, GSK Biologicals and MVI.

## Methods

### Study design

**Figure 1:**
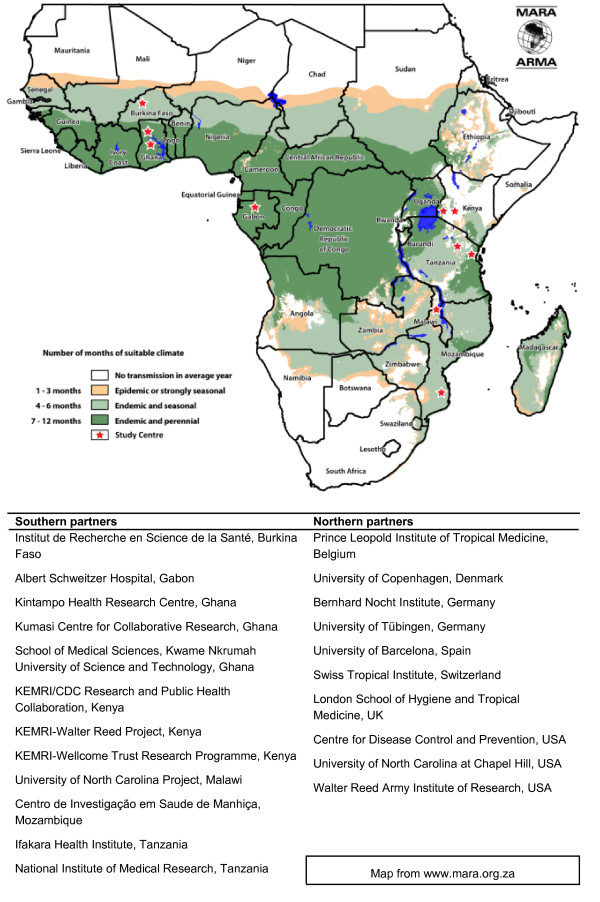
**Study centers and clinical trial partners**.

This is a phase III, randomized, controlled, multicentre, participant- and observer-blind study. Enrolment occurred between May 2009 and February 2011. Follow up is currently on-going at 11 centres covering a wide range of transmission settings in seven countries in sub-Saharan Africa (Figure 1). The study is conducted in accordance with the current Declaration of Helsinki, International Committee on Harmonization Good Clinical Practice guidelines and local rules and regulations of each country. The study is overseen by an Independent Data Monitoring Committee (IDMC), assisted by a Local Safety Monitor at each centre. Approval was obtained from 56 institutional review boards and national Regulatory Authorities. Prior to study inclusion, parents or guardians of all participants provided signed/finger-printed and witnessed informed consent.

**Figure 2:**
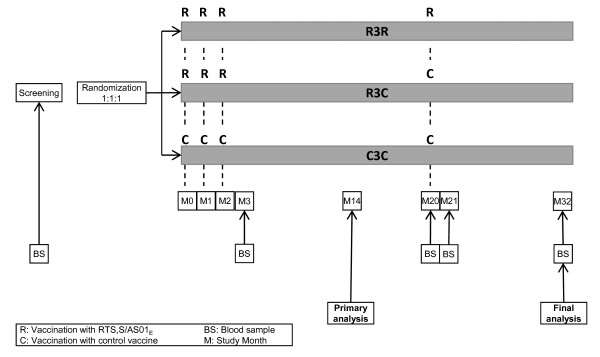
**Study design**.

An overview of the study design is shown in Figure 2. Children were enrolled in two age categories: 6-12 weeks old and 5-17 months old. A minimum of 6,000 children in both age categories (to a total maximum of 16,000 children) have been enrolled and randomized 1:1:1 to one of three study groups for primary and booster vaccination (Table 1). The control vaccine given depends upon the age of the child at enrolment (Table 1). Children in the younger age category receive their primary vaccination course at 6, 10 and 14 weeks of age, in co-administration with the other vaccines usually administered at these EPI visits (Table 1). The specified age range of 6-12 weeks at first vaccination allows for some flexibility in this schedule to align with local guidelines and practice. Bacillus-Calmette-Guérin (BCG) vaccine, neonatal dose of oral polio vaccine (OPV), measles vaccine and yellow fever vaccine are given according to local policy.

**Table 1:** Treatment groups and vaccination schedule

	Primary vaccination: 0, 1, 2 months	Booster vaccination: month 20
**Children 5-17 months of age**		

"R3R" group	RTS,S/AS01	RTS,S/AS01
"R3C" group	RTS,S/AS01	Control (MCC vaccine)
"C3C" group	Control (rabies vaccine)	Control (MCC vaccine)

**Children 6-12 weeks of age**		

"R3R" group	RTS,S/AS01 + DTPwHepB/Hib + OPV	RTS,S/AS01 + OPV
"R3C" group	RTS,S/AS01 + DTPwHepB/Hib + OPV	Control (MCC vaccine) + OPV
"C3C" group	Control (MCC vaccine) + DTPwHepB/Hib + OPV	Control (MCC vaccine) + OPV

MCC: Meningococcal C conjugate vaccine - Meningitec™ (Wyeth), NeisVac-C™ (Baxter) or Menjugate™ (Novartis)

Cell culture rabies vaccine - human diploid cell rabies vaccine (Imovax™, Sanofi Pasteur), purified chick embryo cell culture vaccine (Rabipur™/Rabavert™, Novartis) or purified Vero cell culture rabies vaccine (VeroRab™, Sanofi Pasteur)

DTPwHepB/Hib - Tritanrix HepB™ and Hiberix™ (GSK Biologicals)

OPV: Oral polio vaccine - Polio Sabin™ (GSK Biologicals)

Each child will receive all three doses of rabies vaccine or MCC vaccine from the same manufacturer

Primary immunizations are administered by intramuscular injection into the antero-lateral thigh (children aged 6-12 weeks) or the left deltoid (children aged 5-17 months); all children receive the booster injection in the left deltoid. Neither the study subjects and their parents/guardians nor the study personnel involved in evaluation of the study endpoints are aware of the group allocation of the subjects. Because the study vaccines differ in appearance, the study staff responsible for their preparation and administration is aware of treatment allocation and therefore perform no other role in the trial.

There is no routine testing for HIV infection in this study, HIV tests are performed only if clinically indicated. Voluntary counselling and testing, Highly Active Antiretroviral Therapy (HAART) and Prevention of Mother to Child Transmission (PMCT) are available at all study centres according to national policies.

In accordance with national policies, all centres use artemisinin-based combination therapy (ACT) as first line treatment for malaria cases. The use of insecticide-treated bed nets is optimized at all centres by the close collaboration between research staff and malaria control program managers or through distribution at screening. Other control interventions such as intermittent preventive treatment in infants (IPTi) and indoor residual spraying (IRS) are not currently part of policy in the study areas, but if this changes during the trial, their use will be recorded.

The overall sample size has taken into account the recent description of falling rates of malaria disease in several parts of Africa. To be assured of meeting the primary endpoints, conservative rates of disease were applied in the calculation of sample size and a case driven approach selected [3]. To control for the co-primary endpoint in each of the two age categories, evaluations will be performed at a 2.5% alpha level (Bonferroni correction). Assuming an attack rate in controls of 10/100 children years at risk (cyr), a 12 months follow up period, a true vaccine efficacy of 30% and a drop-out rate of 10% then the sample size of 6000 children in each age category has 90% power to detect a lower limit of the 97.5% CI around estimated VE above 0%. In the event that the attack rate is lower than anticipated, the analysis will be postponed until 450 cases have accumulated. Due to the uncertainty around the rate of severe malaria disease according to the case definitions used in the trial, the total sample size is up to 16000 children and the analysis will be conducted for both age categories pooled when 250 episodes have accumulated. This gives 80% power to detect 30% VE with a lower limit of the 95% CI above 0% or assuming 50% VE 90% power to detect a lower limit of the 95% CI above 25%.

### Study subjects

Inclusion and exclusion criteria are shown in Table 2. The aim was to enrol a broad sample of children representative of the general population. Exclusion criteria have been kept to a minimum to mirror the general population as far as possible whilst minimizing participant safety risk exposure. Children with a history of simple febrile seizure, malnourished children not requiring hospitalization and HIV-infected children (other than those with HIV disease stage 3 or 4 severity as defined by the WHO 2005) were not excluded from study participation.

**Table 2:** Inclusion and exclusion criteria

Inclusion criteria	Male and female children aged 6-12 weeks or 5-17 months at time of first vaccination
	
	Children in 6-12 week age category must be more than 28 days old at screening and must not have received previous vaccination against diphtheria, tetanus, pertussis or *Hemophilus influenzae *type B
**Exclusion criteria**	Acute disease at time of enrolment
	
	Acute or chronic, clinically significant pulmonary, cardiovascular, hepatic or renal functional abnormality
	Major congenital defect
	Malnutrition requiring hospitalization
	Hb ≤8 g/dL with clinical signs of heart failure or severe respiratory distress OR Hb ≤5 g/dL
	Currently meeting WHO criteria for stage III or IV severity HIV disease
	History of allergic reactions, significant IgE-mediated events or anaphylaxis to previous immunizations
	History of allergic disease or reactions likely to exacerbated by any component of the vaccine
	History of a neurological disorder or atypical febrile seizure
	Concurrently participating in another clinical study of a drug or vaccine unlicensed for that indication, except studies aiming to improve treatment or management of severe malaria
	Use of a drug or vaccine unlicensed for that indication other than study vaccines within 30 days preceding the first dose of study vaccine or planned use during the study period
	Previous participation in another malaria vaccine trial
	Receipt of a vaccine within the preceding 7 days
	Other factors that the investigator considers would increase the risk of an adverse outcome or result in incomplete or poor quality data

### Study vaccines

The candidate malaria vaccine is RTS,S/AS01 (GSK Biologicals, Rixensart, Belgium). The RTS,S antigen is a hybrid recombinant protein consisting of the *P. falciparum *circumsporozoite (CS) protein central tandem repeat and carboxy-terminal regions fused to the amino-terminus of the S antigen of hepatitis B virus (HBsAg). The vaccine is formulated with the AS01 Adjuvant System.

The choice of comparator vaccines was guided by the need to offer potential benefit to the control group without compromising the evaluation of study endpoints. The pros and cons of a number of options were debated and consensus reached by the CTPC after taking into account regional epidemiology and EPI programs. Rabies vaccine was chosen as the control for the 5-17 month age category because of the high burden of rabies across all of sub-Saharan Africa, its high fatality rate and the particular risk to children [17-19]. Rabies vaccine has been evaluated according to several different vaccination schedules, and the 0, 1, 2-month schedule used in this trial is expected to produce acceptable antibody titers and provide protection. Rabies vaccine was not appropriate for children in the 6-12 week age category because co-administration with EPI antigens has not been evaluated. For the 6-12 week age group, consideration was given to vaccines against *S. pneumoniae*, which is a common cause of pneumonia in children in Africa. The reasons for not selecting pneumococcal vaccines were that they were expected to be implemented as policy in some countries prior to the enrolment and so would not provide additional benefit. In addition, there is a poorly understood interaction between malaria and pneumococcal infections. In paediatric hospital admissions, pneumonia and malaria co-occur more often than expected by chance [20]. This may be due to the overlapping clinical symptoms and signs of pneumonia and malaria, or the immunosuppressive effect of malaria infection on pneumococcal pneumonia [21]. The careful characterization of both malaria and pneumonia in this trial will also allow the study of the effect of malaria control on the incidence of pneumococcal disease [22]. Meningococcal disease in Africa is most commonly due to serogroup A, however there currently is no meningococcal A vaccine licensed for use in infants. Meningococcal C conjugate vaccine was chosen because it is acceptably safe and immunogenic when administered according to a 0, 1, 2-month schedule, can be safely co-administered with the other vaccines and will not compromise the analysis of the study endpoints. Although meningitis C is not common in sub-Saharan Africa outbreaks have been reported [23-25] and, therefore, the vaccine may provide some benefit to study subjects.

### Endpoint data collection

Clinical malaria cases are detected through passive surveillance at local health facilities. A blood sample for evaluation of malaria parasites is taken from all children with axillary temperature of ≥37.5°C or those reported to have had a fever within 24 hours of presentation. All subjects attending hospital emergency departments in the study areas are evaluated as potential cases of severe malaria following an algorithm, and case assessment is standardized across centers [22]. The algorithm also allows identification of cases of anaemia, sepsis and pneumonia.

Two cross-sectional surveys will be conducted at study months 20 and 32 to assess vaccine efficacy against prevalent parasitaemia and anaemia. Data will be collected on potential covariates which may be included in the analysis of efficacy. These are bed net usage by direct observation, application of IRS, administered doses of IPTi, distance from nearest inpatient health facility, distance from nearest outpatient health facility, pneumococcal/Hib vaccination status, ethnicity, anthropometric measurements and feeding history.

Full quality systems are in place for all laboratory tests in the trial and these are described in a companion paper [26]. Anti-CS and anti-HB antibody titers are measured at all blood sampling time points in a subset of children from both age categories at all sites (See Figure 2 for blood sampling time points). In addition, a nested case control study will evaluate the association between CS-antibody response and protection against malarial disease. In a safety and immunogenicity trial of RTS,S/AS01 co-administered with EPI vaccines, pre-defined non-inferiority criteria compared to control were met for all the DTPwHepB/Hib+OPV, measles and yellow fever antigens, with the exception of polio 3 viruses when RTS,S/AS01 was administered at 0, 1, 2-months [27]. Although a post-hoc analysis showed that differences were explained by pre-vaccination titres, additional data will be collected in this phase III study. Titres will be assessed in a subset of infants in the 6-12 week age category at each site at three time points: study start, one month post primary vaccination, and one month post OPV booster vaccination.

Serious adverse events (SAE) are collected for all subjects for the entire study period. Completeness of SAE reporting is strengthened by monthly visits of field workers to the children's homes. SAEs are defined as AEs resulting in death, which are life-threatening or require hospitalization or prolongation of existing hospitalization or those that result in disability or incapacity. Unsolicited AEs occurring during the 30 days after each vaccine dose and solicited AEs occurring during the seven days after each vaccine dose are collected for the first 200 children enrolled in each age category at each centre. In the remainder of children, only AEs that are considered to be related to vaccination or those resulting in study withdrawal are recorded. Investigators will grade all AEs and SAEs as mild, moderate or severe based on a scale of interference with normal daily activities, and assess the relationship to vaccination.

Seizures occurring within 30 days of vaccination are also required to be reported as SAEs. For seizures occurring within seven days of vaccination, an analysis will be performed based on the Brighton Collaborations guidelines, which captures the features of the seizure and classifies the level of diagnostic certainty [28]. In the first 200 subjects enrolled at each site in the six to 12 weeks age category an analysis of rashes and mucocutaneous diseases within 30 days of vaccination will be performed based on the Brighton Collaboration Guidelines [29]. Due to a theoretical concern that the use of new adjuvanted vaccines may interfere with immunological self-tolerance, regulatory authorities have requested data collection on immune-mediated diseases (IMD). Therefore, all IMDs are reported as SAEs for all subjects over the entire study period. Diagnostic support at a referral laboratory is provided.

Solicited local (injection site) AEs recorded are pain, swelling and redness; grading of symptoms is on a scale of 0-3. Solicited general AEs recorded are drowsiness, fever, irritability/fussiness and loss of appetite; intensity of symptoms (except fever) are graded on a scale of 0-3 based on interference with normal daily activities; fever is defined as axillary temperature ≥37.5°C. Methods have been fully described previously [30].

Verbal autopsies are carried out for all children who die outside a health facility to ascribe the cause of death. The questionnaire used is based on the INDEPTH standard and adapted to be locally appropriate [31]. At study end, all forms will be read by a central panel to attribute cause of death. As a general health indicator, growth is monitored throughout the study according to standardized methods. The length (<2 years of age) and height (≥2 years of age), weight and mid-upper arm circumference are measured at first vaccination and at study months 3, 20 and 32.

Safety and immunogenicity will be described in the special sub-populations of malnourished and HIV-infected children. Weight at enrolment will be used to determine a subset of children who are low weight for age (weight for age z-score ≤-2) and very low weight for age (weight for age z-score ≤-3). HIV infections known at enrolment or diagnosed during the trial are recorded.

### Study objectives and case definitions

#### Primary efficacy objectives

The co-primary objectives of the study are efficacy over 1 year post-dose 3 against clinical malaria when primary immunization starts at: (1) 6-12 weeks of age, with co-administration of DTPwHepB/Hib and OPV antigens; (2) 5-17 months of age (Table 3). Clinical malaria was selected as the primary endpoint for this trial. There is an enormous burden of disease in sub-Saharan Africa associated with clinical malaria that puts substantial demands on the health services of these countries [32]. It is a serious condition with approximately 2% of cases progressing to severe and life threatening forms of the disease [33]. Severe malaria was not selected as the primary endpoint of the trial because of uncertainty surrounding rates of severe disease. Indeed, malaria incidence appears to be falling in areas of Africa where effective malaria control measures, such as insecticide-treated bed nets and first-line treatment with ACT, have been implemented [3]. A vaccine that is effective against clinical malaria is likely to be at least as efficacious against severe disease.

**Table 3:** Study objectives

**Efficacy**	Efficacy against clinical malaria over 1 year in children aged 6-12 weeks at first vaccination (co-administration of DTPwHepB/Hib)^1^ Efficacy against clinical malaria over 1 year in children aged 5-17 months at first vaccination^1^ Efficacy against severe malaria Prevention of anaemia (incident severe anaemia; prevalent moderate and severe anaemia) Prevention of malaria hospitalization Evolution over time of efficacy following the primary vaccination course Additional benefit of a booster dose Efficacy in different transmission settings Efficacy against parasite prevalence Efficacy against other serious illnesses (medical hospitalization, sepsis and pneumonia) Efficacy against fatal malaria and all-cause mortality Effect on growth Gender-specific efficacy^2^
**Immunogenicity**	Immunogenicity of a primary vaccination course Immunogenicity of a booster dose Immunological correlates of protection Immunogenicity of the oral polio vaccine when co-administered with RTS,S/AS01
**Safety**	Safety of a primary vaccination course Safety of a booster dose
**Special populations**	Immunogenicity and safety in HIV-infected children Immunogenicity and safety in low weight for age children

^1 ^Represent the primary objectives

^2 ^If important differences in the co-primary objectives are observed between boys and girls, all primary and secondary efficacy and immunogenicity endpoints will be presented separately for boys and girls

The primary case definition of clinical malaria upon which the primary endpoint will be assessed is presented in Table 4. One of the criteria of the case definition is that the child is unwell and brought to a health care facility. This is to ensure that the cases of malaria are representative of the severity of cases using health services and is a measure of public health relevance. It is likely that most cases of clinical malaria that occur in the community will present to healthcare facilities because all children in the study areas have reasonable access to healthcare, and if any costs are incurred, these are reimbursed by the study.

**Table 4:** Case definition of clinical malaria

Criterion	Primary definition	Secondary definition 1	Secondary definition 2	Secondary definition 3
Threshold of *P. falciparum *asexual parasitaemia	>5,000 parasites/μL	>0 parasites/μL	>500 parasites/μL	>20,000 parasites/μL

Fever	axillary temperature ≥37.5°C	axillary temperature ≥37.5°Cor history of fever within 24 h of presentation	axillary temperature ≥37.5°C	axillary temperature ≥37.5°C)

Case detection	Child is unwell and brought to healthcare facility	Child is unwell and brought to healthcare facility	Child is unwell and brought to healthcare facility	Child is unwell and brought to healthcare facility

Other	**OR** Meets primary case definition of severe malaria^1^			

^1 ^Refer to [22] for primary definition of severe malaria

Another criterion of the definition is a parasite density threshold. This has been added to increase the specificity of the case definition. Achieving a balance between specificity and sensitivity is a major challenge in defining endpoints in malaria vaccine trials. Low specificity means that vaccine efficacy is likely to be underestimated, whereas low sensitivity means that the power of the study will be reduced [34]. Achieving adequate specificity in the case definition of clinical malaria is difficult, as the symptoms of malaria overlap with those of many other common febrile childhood illnesses. It is well recognized that as parasite density increases, the likelihood that symptoms are caused by *P. falciparum *infection also increases. A widely-used methodology in malaria research is applied to calculate the specificity and sensitivity of clinical case definitions according to parasite density threshold values [35]. A single parasite density threshold of 5,000 parasites/μL is employed across all centres for the primary endpoint to support pooling of data. This threshold was based on data from previous studies [35-40], and provides a minimum specificity of 80% for all transmission settings and age categories in this trial. By adding the requirement for fever and a parasite density threshold we adhere to the accepted practice to evaluate malaria disease interventions. However, in the evaluation of IPTi, using case definitions with varying parasite density thresholds has not yielded the expected increase in specificity reflected in the estimate of effect [41]. In the case of an intervention that is equally protective against symptomatic parasitaemia and asymptomatic parasitaemia less specific definitions for malaria disease may not significantly impact efficacy estimates. This appears to be the case for this pre-erythrocytic vaccine [14,42].

The principal analysis for the determination of the primary endpoint is protection against first or only episodes of malaria using a hazard ratio estimated from Cox regression model. This will be adjusted for centres to control for differences in malaria transmission between centres. The statistical methodology is further discussed in the companion paper [43].

#### Secondary efficacy objectives

A wide range of secondary objectives are included in this trial to support a full evaluation of the potential public health impact of the vaccine and to aid policy and implementation decisions. Secondary efficacy objectives are shown in Table 3. The trial will look at the full spectrum of disease manifestations from clinical malaria to severe and fatal disease. Case definitions for secondary endpoints relating to disease manifestations are shown in Tables 4 and 5, with the exception of severe malaria. Severe malaria is a key endpoint in this study and is described in further detail in a companion paper [22].

**Table 5:** Case definitions of secondary efficacy endpoints

	Definition 1	Other definitions
**Incident severe anaemia^1^**	Hb <5.0 g/dL identified on morbidity surveillance in association with *P falciparum *parasitaemia >5000 parasites/μL	Hb <5.0 g/dL identified on morbidity surveillance PLUS 1. *P falciparum *parasitaemia >0 parasites/μL OR 2. No parasitaemia

**Prevalent anaemia^1^**	Hb <5.0 g/dL identified at cross sectional survey	Hb <8.0 g/dL identified at cross sectional survey

**Malaria hospitalization**	Medical hospitalisation^2 ^in association with *P falciparum *parasitaemia >5000 parasites/μL	*P falciparum *infection is sole or major cause of hospitalization on investigators' clinical judgement

**All hospitalization**	Medical hospitalization^2^	

**Bacteremia/Sepsis**	Positive blood culture^7^	

**Pneumonia**	Cough or difficulty breathing (on history) Tachypnoea (≥50 breaths per minute in children <1 year, ≥40 breaths per minute in children ≥1 year) Lower chest wall in-drawing	As definition 1, PLUS 1. Chest X-ray consolidation or pleural effusion on a chest X-ray taken within 72 h of admission OR 2. Chest X-ray consolidation or pleural effusion or other infiltrates on a chest X-ray taken within 72 h of admission OR 3. Oxygen saturation <90%

**Fatal malaria**	Fatal case of severe malaria according to primary case definition 3,4	Fatal case of severe malaria according to secondary definitions 3,4

**All-cause mortality**	Death due to any cause5	Death due to medical cause5,6

^1 ^Severe anaemia is defined as Hb <8 g/dL and very severe anaemia is defined as Hb <5 g/dL according to WHO/IVR report (WHO/IVR Malaria Vaccine Advisory Committee meeting 2004)

^2 ^Excludes planned, surgical and trauma related admissions

^3 ^Refer to [23] for primary and secondary definitions of severe malaria

^4 ^Restricted to children fully evaluated as inpatients and excludes diagnoses made by verbal autopsy

^5 ^Includes deaths in hospital and in the community

^6 ^Excludes trauma, which may be diagnosed by verbal autopsy

^7 ^A blood culture taken within 72 h of admission is considered positive if: a definite pathogen is isolated or a bacteria that could be either a pathogen or a contaminant is isolated within 48 hours of incubation and is considered clinically to be a likely pathogen [23].

The case definitions have been selected to be consistent with usual practice wherever possible to allow generalizability of trial findings and comparability with other interventions. Where applicable for fuller interpretation of the data, multiple case definitions for a given endpoint have been used. For example, three secondary definitions of clinical malaria will be analysed (Table 4). The first is highly sensitive and includes detection of any parasite level, plus a history of fever, and is not limited to measured fever at presentation. This definition mirrors the children who are treated under current WHO guidelines and will be important for consideration of the impact of the vaccine on disease burden and in health economic analyses. The second case definition is designed for the analysis of infants; the lower parasite density threshold of >500 parasites/μL may be appropriate for this age group [36,40]. The third definition, with a high parasite density threshold, is included because it is highly specific.

This study aims to characterize the potential indirect benefits of malaria control through vaccination using the complete morbidity data set collected. Trials with insecticide-treated bed nets have shown a reduction in all-cause mortality that is not solely accounted for by malaria-specific mortality characterized on verbal autopsy [44,45]. There was an indication of indirect benefits associated with RTS,S/AS vaccination in phase II trials; in one study, the overall trend of fewer SAEs in vaccine recipients was only partly accounted for by malaria events [14], and in another study, pneumonia hospitalization was less common in vaccine recipients [13]. Specifically, the possible vaccine benefit on bacteraemia/sepsis and pneumonia will be investigated. The evaluation and definition of pneumonia is based on the extensive methodological and case definition work done to support paediatric pneumococcal conjugate vaccine trials [46].

How the spectrum of clinical benefits provided by vaccination evolves with time will be critically important for policy decisions. This trial will provide information up to 2.5 years after a primary vaccination course. The evaluation of efficacy over time is complicated; children progressively acquire natural immunity as they age and therefore whilst the biological action of the vaccine may persist unchanged the vaccine efficacy that is measured relative to control will fall. Inclusion of a group that will receive a booster dose allows the potential benefit of a primary course plus booster to be measured against both the comparator and against the primary course alone.

To support extrapolation of trial results to other regions, data will be generated from a number of diverse malaria transmission settings; the participating centers are shown superimposed on the Mapping Malaria Risk in Africa (MARA) map of malaria risk in Africa in Figure 1. Site-specific estimates of efficacy against clinical malaria will be produced (this is further detailed in a companion paper [43]).

## Conclusion

This pivotal phase III efficacy study of the RTS,S/AS01 candidate malaria vaccine in African children was designed and implemented by the Clinical Trials Partnership Committee. The study design is in full accord with the conclusions of the WHO expert panel on the measurement of malaria vaccine efficacy in phase III clinical trials [47]. The design described here evolved over approximately five years of planning, through collaboration between, and with input from, scientists, regulators, and policy-makers. The study will provide efficacy and safety data to fulfil regulatory requirements, and will supply data on a broad range of disease endpoints that will allow evaluation of the public health impact of the vaccine. Data from the primary analysis of clinical malaria one year after completion of the primary immunization course of children aged 5-17 months are expected to be available in 2012. At the end of the study in 2015, data will be available on a wide range of disease endpoints, as well as information on the evolution of efficacy over time, the potential added benefit of a booster dose and long-term safety.

## Competing interests

AL, JV, MLi, OOA, CC, WRB, LV are employees of GlaxoSmithKline Biologicals. AL, JV, and WRB hold shares in GSK, WRB holds several patents, including one for RTS,S/AS.

GSK and all participating institutions declare to have received a grant and travel funds from PATH-MVI for/relating to the clinical trial described in this manuscript. Employees of PATH-MVI (BS, CL, AB, FD, SF, CH, MM, JM, CP, DP, and MS) report their institute received a grant by the Bill and Melinda Gates foundation for this trial. All GSK employees, SA, PB, and PN declare their institution has received grants or has grants pending from PATH-MVI for previous clinical trials. CSS, JFT declare no potential conflicts of interest.

## Authors' contributions

All authors and members of the Clinical Trials Partnership Committee contributed to the design of the phase III trial through discussions and document review. AL led the writing of the manuscript coordinating the incorporation of all reviewer comments. SOA, SA, EM, and PN lead the review process by chairing the CTPC and leading the scientific debate and review process around trial design. AL, JV, OOA, CC, ML, and WR wrote the study protocol from which the tables and graphics are drawn. BS and CL coordinated the input of the MVI team. All authors read and approved the final manuscript.
